# Inherent safety risks of guidelines derived from “cause-agnostic” randomized controlled trials: a review

**DOI:** 10.1186/s13037-026-00486-y

**Published:** 2026-04-16

**Authors:** Lawrence Lynn

**Affiliations:** Doctors Hospital. OhioHealth, Columbus, OH USA

## Abstract

Physicians rely on guidelines derived from randomized controlled trials (RCTs) to determine whether interventions are safe and effective for specific diseases or causal mechanisms. In critical care, however, well-powered RCTs demonstrating clear evidence of benefit and subsequently incorporated into clinical guidelines have repeatedly been abandoned after causing harm. This strong evidence of benefit from robust RCTs or meta-analyses followed by unexpected harm during clinical application, generates an “evidence deployment paradox”, which has been attributed to unavoidable biological heterogeneity rather than to structural flaws in trial design. Recent analysis has shifted attention to the architecture of the RCT itself, indicating that the paradox arises from a mathematical set-mixing problem introduced by a modification of the original RCT model when the field adopted a trial design in which enrollment was decoupled from causal diagnosis or the targeted causal mechanism and replaced by consensus-derived, disease- and cause-agnostic triage thresholds. The triage selects broadly shifting mixtures of diseases with potentially different treatment effects. We term this design the cause-agnostic randomized controlled trial. Although cause-agnostic RCT can generate internally valid estimates within trial participants, their results may paradoxically be unsafe to incorporate into clinical guidelines, even when applied in the same hospitals and using the same entry criteria as the source trials. Structural causal modeling provides tools to mitigate this evidence-deployment paradox. Such modeling makes explicit the causal assumptions underlying enrollment and can identify when, and to what extent, a trial has deviated from cause-and-effect logic of the original randomized trial design.

**Supplementary Information:**

The online version contains supplementary material available at 10.1186/s13037-026-00486-y.

## Introduction

This review is a companion of a more comprehensive mathematical analysis [1] and the interested reader is referred to that review for mathematical context. The present article focuses on presenting a taxonomy as well as the public safety considerations of guidelines derived from modern critical care trials.

The randomized controlled trial (RCT) was developed to answer causal questions in medicine. As introduced by Fisher [2], randomization was a procedural tool to distribute unknown differences evenly between treated and untreated groups, originally applied to agricultural experiments in which experimental units represented a single causal entity. Building on this framework, Bradford Hill [3, 4] emphasized that clinical RCTs must define diagnostic or clinical criteria that select participants sharing the causal disease mechanism targeted by the intervention. When treatment effects were robust and causally coherent, trial entry criteria and interventions could be cautiously transported into bedside protocols under expert clinical judgment.

We refer to this original design as the causal-integrity randomized controlled trial (CIR), in which treatment, diagnostic selection, and disease mechanism are explicitly aligned. In this framework, the RCT functions as one component of a broader causal reasoning process, generating a scaffold of transportable knowledge. Trials that preserve this alignment retain causal integrity and can safely inform clinical protocols. The CIR, the original species of RCT, was one of the major scientific advances of the twentieth century. However, within a few decades, a streamlined alternative emerged that decoupled enrollment from causal diagnosis and came to dominate parts of clinical science.

## Emergence of a new RCT “species”

Over subsequent decades, pressure to apply RCTs to poorly defined clinical conditions, particularly in critical care, fostered the belief that entry criteria selective for a shared causal disease mechanism were unnecessary. Instead, it came to be assumed that randomization within a population defined solely by disease- and cause-agnostic triage criteria (for example, clinical presentation plus non-specific laboratory thresholds) was sufficient to support causal inference and trial validity [1]. Under this view, an RCT could estimate a treatment effect in any broadly specified population. We term this modified design the cause-agnostic randomized controlled trial (CAR).

This shift did more than simplify trial conduct; it fundamentally altered the causal question, from whether a treatment benefits patients with a specific cause or disease mechanism to whether it produces an average effect in a heterogeneous, triaged population. Within the cause agnostic RCT framework, demonstration of a population-level effect was implicitly treated as proof of causation, rendering mechanistic specificity optional. This represents a regression to pragmatic empiricism, echoing William James’s assertion that “Truth happens to an idea. It becomes true, is made true by events. Its verity is in fact an event, a process: the process namely of its verifying itself, its veri-fication.” [5]. In the field of critical care the assumption was that truth would someday happen to the decade’s old ideas of using a consensus set of causal disease agnostic threshold to study the synthetic syndromes of adult respiratory distress syndrome (ARDS) and sepsis.

Therefore, the idea of using a triage set of thresholds as trial entry criteria was tested in experience and “became true” because it worked to streamline trials. Yet, this was not the standard by which “truth” should have been assessed. The most relevant “truth” was that trial-to-clinical population matching based on broad, non-specific and disease agnostic triage criteria increased the risk of guidelines derived from unsafe protocol transport in complex clinical environments.

While such pragmatism may be acceptable in exploratory science, its use in guideline protocol-generating clinical trials without a causal anchor obscures the risk that statistically valid results will yield clinically unsafe guidance due to causal misalignment. This risk was clearly demonstrated by prior reversed sepsis and ARDS protocols [1] and again during the COVID-19 pandemic.

## Unjustified extrapolation of cause-agnostic trials: The “false RCT transport”

False RCT transport occurs when population-based eligibility criteria from a cause-agnostic randomized controlled trial (CAR) are repurposed as guidelines or treatment-triggering rules in clinical care despite the absence or underrepresentation of the causal disease mechanisms in the source trial. This design-level failure converts internally valid trial results into unsafe bedside protocols when causal alignment is lacking.

During the COVID-19 pandemic, Acute Respiratory Distress Syndrome Network-derived FiO₂/PEEP Table [6], supported by meta-analyses of synthetic syndrome, enrolled cause-agnostic RCTs [1, 7], were rapidly implemented, and high-PEEP strategies were promoted as “strong” ventilator guidance [8]. Notably, this designation of evidentiary strength persisted despite COVID-19 representing a novel disease entity that was entirely absent from the source trials, reflecting the assumption, implicit in synthetic-syndrome reasoning, that fulfillment of consensus criteria alone is sufficient to justify protocol transport. This is the 50 year old symbol–mechanism substitution fallacy that underlies much of critical care RCT science today.

The absence of COVID-19 pneumonia from the source meta-analysis [7] was only part of the problem. More fundamentally, the meta-analysis represented the extreme endpoint, indeed a prototypic example, of institutionalized pathological synthetic-syndrome science. Multiple cause-agnostic randomized controlled trials, each individually unsafe for transport [1], were aggregated as if they were commensurable. In doing so, complex relational physiology, including gas exchange dynamics, lung mechanics, and wide heterogeneity in disease processes and pathogens, was collapsed into a single pooled estimate. The resulting output could not plausibly preserve causal meaning and therefore could not be expected to yield clinically reliable guidance.

The assertion that “strong” evidence existed for high-PEEP tables exposes the characteristic reasoning of cause-agnostic trialism: the belief that complex physiology and distinct disease processes can be simply discounted and collapsed by disease and cause-agonistic threshold consensus triage, RCTs, and meta-analyses. This is the fundamental epistemic error of cause-agnostic critical-care science which was exposed by the COVID-19 pandemic.

It can be difficult for scientists unfamiliar with synthetic-syndrome reasoning to understand how “strong” evidence is claimed at the onset for the treatment of a novel disease. Within the synthetic-syndrome framework, however, this conclusion follows naturally. Once a set of consensus-derived criteria is defined, any condition that satisfies those criteria is treated as causally equivalent to all others that do so, even if the condition itself did not exist, or was not recognized, when the consensus was established. Evidence is therefore attributed to the criteria, not to the disease process itself.

For this reason, once COVID-19 pneumonia met ARDS criteria, trial-derived ventilation strategies were assumed to represent “strong” evidence and were transported wholesale into COVID-19 care, without the warning to clinicians that such transport might be unsafe because the experts assumed it was safe. Although the use of this treatment was defensible early in the pandemic, the effect of assuming it was strong evidence meant that protocol failure detection systems were not perceived as needed.

This reasoning exemplifies synthetic science [1]. Using disease- and cause-agnostic triage thresholds, a synthetic syndrome, a “disease equivalent” is constructed to enable streamlined randomized controlled trials. Although all formal elements of experimentation are present, including randomization, outcome measurement, and statistical analysis [9], no specific disease entity or causal mechanism is studied. As a result, trial-derived protocols are assumed to be transportable to any condition meeting the triage criteria, including diseases that did not exist when the trials were conducted, despite the absence of causal grounding.

Predictably, this approach failed to produce safe clinical protocols. During COVID-19, mortality among invasively ventilated patients proved profound and physiologically implausible [10, 11], leading bedside clinicians, operating without formal protocol-effect surveillance or failure-mode monitoring [12], to abandon these protocols. This reversal was not driven by new randomized evidence, but by real-world outcomes incompatible with the assumptions underlying the transported trial guidance.

This episode represents a real-world falsification of the prevailing epistemology of critical care synthetic syndrome science and of the assumed transportability of cause-agnostic RCT (CAR)–derived protocols. But, in hindsight, this episode constitutes just one instance of a long standing system-level patient-safety failure arising from the large-scale deployment of protocols derived from an NIH-institutionalized variant of the randomized controlled trial that lacked safeguards against false RCT transport. Similar reversals across critical care are well documented [1, 13], indicating that this dominant failure mode has been present for decades and lies not in trial execution or statistical analysis, but upstream in trial design, specifically, in the replacement of causal-integrity RCT (CIR) architecture with structurally non-transportable cause agnostic RCT designs.

## Cause-agnostic vs. causal-integrity trials

The cause agnostic RCT and causal-integrity RCT species exist along a spectrum. For pedagogic clarity, we introduce a binary design taxonomy:

## Cause-agnostic RCT (CAR)

- Enrollment is conditioned on disease and cause-agnostic population triage (as by consensus based, but disease and cause agnostic, lab value thresholds.)
- Multiple distinct upstream causes may be aggregated prior to randomization.
- Entry criteria are purely population based and lack reliable safe transportability to clinical protocols.
- Meticulous attention to internal validity.
- High public safety risk, design very carefully with prevention of false RCT transport in mind. Have protocol failure detection system in place.

## Causal-integrity RCT (CIR)

- Enrollment criteria includes a diagnostic measurement, historical event or definitive clinical finding which assures that substantially all participants included in the RCT have the cause, disease or harm prevention mechanism which is targeted by treatment in the trial.
- Entry criteria are transportable to clinical protocols selecting patients with the same target by applying the same or equivalent selection strategy used in the trial.
- Meticulous attention to internal validity.
- Low public safety risk of false RCT transport.

Since the cause agnostic RCT is triage-population-selective rather than causal-target-selective, the trial’s disease and cause agnostic population triage alone are subsequently embedded into clinical protocols and transported to the bedside as guidelines. As a result, this is a population based RCT (a cause agnostic RCT) so the treatment effect becomes inseparable from the population triage, which must therefore be capable of reliably reproducing a clinical patient population with the same unknown underlying disease mechanism as that blindly studied in the source trial. This assumption is pivotal for safe transport to the clinical protocol but is often false.

In practice, this approach amounts to an uncontrolled causal gamble: a treatment was tested in a triage defined population with the hope that a positive signal would reveal a true and transportable treatment effect. The central danger was not merely trial failure, but apparent success. Because the trial population was defined by nonspecific, threshold-based criteria that could not reliably reproduce the same underlying disease mechanisms, a positive result could be falsely transported into clinical protocols. In this way, apparent efficacy is converted into a latent public-safety hazard, exposing the public to treatments that had never been causally validated for their underlying pathology.

The emergence of the cause-agnostic randomized controlled trial in critical care was not accidental. Early sepsis task forces introduced cause agnostic RCT logic to harmonize trials of the synthetic syndrome of sepsis [14], but durable institutionalization occurred through large NIH-funded critical-care networks, most prominently the Acute Respiratory Distress Syndrome Network (ARDSNet), which established a centralized template for syndrome-gated RCTs [15].

The Prevention and Early Treatment of Acute Lung Injury (PETAL) Network completed this transition by explicitly broadening eligibility to undifferentiated acute respiratory failure and replacing causally selective diagnostic gates with pragmatic triage criteria optimized for speed, scalability, and enrollment efficiency. In doing so, PETAL embedded causal agnosticism not as a limitation, but as a governing design principle, structurally optimizing trials for rapid, large-scale production under centralized NIH governance while unknowingly sacrificing safety of transportability [16].

As a result, synthetic syndromes became the organizing units for trials, guidelines, and quality metrics, independent of causal disease mechanisms. Once centrally governed syndromic triage replaced causally selective enrollment, the clinical trial enterprise became optimized for high-volume trial production rather than causal explanation or cumulative knowledge building. Institutionalized cause agnostic RCT production therefore rendered false RCT transport at scale not an aberration, but a predictable output of the NIH research system itself (Table 1).

**Table 1:** NIH-affiliated programs institutionalizing Cause-agnostic RCT (CAR) governance

Program / Network	NIH Institute(s)	Dates (Launch → Status)	Role in CAR Institutionalization
ARDSNet	National Heart, Lung, and Blood Institute (NHLBI)	1994 → ~2014 (core network era)	Established centralized syndrome-based RCTs using physiologic thresholds (e.g., PaO₂/FiO₂) as enrollment gates, normalizing protocolized interventions without examining causal specificity or transportability. Established the CAR governance template. [15]
PETAL Network	National Heart, Lung, and Blood Institute (NHLBI)	2014 → present	Expanded enrollment from ARDS to undifferentiated acute respiratory failure, adopting pragmatic cause-agnostic eligibility prior to etiologic clarification and formalizing CAR logic as a design strategy. [16]
REMAP-CAP	National Institute of Allergy and Infectious Diseases (NIAID)	2015 → present (pandemic expansion 2020– )	Adaptive platform trials enrolling populations by triage under synthetic syndrome gates. Briefly shifted to CIR for testing corticosteroids for COVID pneumonia [17]. Returned to Petty-Bone CAR research studying the synthetic syndrome of CAP [18].

Causal-integrity randomized controlled trials (CIRs) and cause-agnostic randomized controlled trials (CARs) define opposite ends of a methodological spectrum, rather than discrete categories. Along this spectrum, trials differ in the degree of causal alignment between the treatment, the enrollment criteria, and the population studied.

At one end are pure causal-integrity RCT, in which treatment targets a specific causal disease mechanism and enrollment is anchored by diagnostic criteria that reliably select that mechanism. Randomization then balances residual variation, and trial entry criteria can be safely transported into bedside protocols using the same diagnostic logic.

At the opposite end are pure cause agnostic RCTs, in which treatment effects are estimated in populations defined solely by syndromic or triage-based criteria, without selection for a shared causal mechanism. In this setting, observed treatment effects are inseparable from the population triage itself, and transportability rests on the untested assumption that future patients selected by the same criteria share the relevant causal structure. A representative example is the “Petty-Bone RCT mimic” [1].

Most contemporary RCTs fall between these extremes. Although such trials may achieve internal validity, their position along the cause agnostic RCT –causal integrity RCT spectrum determines their transport risk: as causal alignment weakens, the safety of repurposing trial entry criteria as treatment-triggering rules in clinical care progressively declines.

If we examine past trials we can discover their relative position on the cause agnostic RCT–causal integrity RCT spectrum.

**Table 2:** Comparison of Pure Cause-Agnostic RCTs (CAR) vs. More Causal-Integrity RCTs (“CIR-like”)

Trial / Intervention	Presence of Shared Causal Mechanism	Intervention–Mechanism Alignment		Outcome in Practice
Aspirin (sepsis trials) [19]	Absent – no universal platelet-mediated pathology	Weak; anti-platelet effect not linked to a common causal pathway		Neutral or negative; no durable protocol adoption
Activated Protein C [20,21] (drotrecogin alfa) – sepsis	Absent – heterogeneous inflammatory and coagulation states	Weak; presumed benefit not consistently present		Initially adopted, later withdrawn for lack of benefit and harm
Neuromuscular Blockade (NMB) [22.^23^]– ARDS	Absent / inconsistent – no universal injurious effort or dyssynchrony	Weak; paralysis as proxy control rather than causal target		Initially promoted, later abandoned as routine therapy
ANDROMEDA-SHOCK (CRT- vs. lactate-guided resuscitation) [24]	Present in most –Shock associated risk of tissue hypoperfusion	Moderately strong; CRT reflects real-time tissue perfusion state		physiologic targeting adopted selectively, not protocolized
PEEP–FiO₂ Tables (ALVEOLI / EXPRESS / LOVS) Meta-analysis [25–27, 7] – ARDS	Absent – recruitability and high lung elasticity not uniformly present	Very Weak; assumes shared recruitability, overdistension vulnerability, and P/F pathophysiology		De-protocolized after COVID ventilator protocol failure; now clinician-driven
ARDSNet ARMA (Low Tidal Volume Ventilation) [28]	Present in most – reduced functional lung volume → strain vulnerability	Strong; low tidal volume directly limits over-distension injury		Durable principle: avoid gross over-distension
PROSEVA (Prone Positioning in Severe ARDS) [29]	Present in most – gravity-dependent collapse and stress heterogeneity	Strong; proning redistributes stress and perfusion		Durable benefit in severe ARDS

As the Table 2 shows “intervention–mechanism alignment” is a pivotal feature to consider and it may occur upstream at the diagnosis (trial gate level) or downstream at a convergence point pathophysiology. This is why knowledge of pathophysiology is pivotal.

Although the Acute Respiratory Distress Syndrome Network Lower Tidal Volume Ventilation Trial (ARMA) employed a broad synthetic-syndrome enrollment gate, its treatment target, mitigation of high tidal volume induced injury, reflected a downstream pathophysiologic convergence shared across ARDS etiologies when functional alveolar volume is reduced. Diverse diseases meeting ARDS criteria converge on a common mechanical vulnerability: a diminished aerated lung exposed to excessive strain. This convergence explains why the ARMA trial [28], despite cause-agnostic aggregation, aligns more closely with a causal-integrity RCT. By constraining tidal volume or driving pressure, the intervention targeted a universal physical mechanism rather than the ARDS synthetic syndrome itself. In causal terms, multiple upstream disease processes flowed through a syndrome-based enrollment threshold into a shared downstream mechanical vulnerability that constituted the true treatment target.

A similar mechanism-anchored rationale explains the durability of prone-positioning trials, most notably in the Prone Positioning in Severe Acute Respiratory Distress Syndrome PROSEVA [29], which targeted gravity-dependent stress concentration, dependent atelectasis, and ventilation–perfusion mismatch, mechanical effects recurring across ARDS etiologies when lungs become heavy, heterogeneous, and regionally perfused. In both trials, residual transport risk remained because enrollment criteria did not directly measure the mechanistic targets; however, a sufficient fraction of enrolled patients expressed the relevant vulnerability, preserving a coherent intervention–mechanism–outcome pathway.

Critically, the success of these mechanism-anchored trials [28, 29] was misinterpreted as evidence that broadly gated, cause-agnostic RCTs were generally valid. This inference error enabled the expansion of cause agnostic logic to pharmacologic trials [19–23] and FIO2–PEEP–based trials [25–27], where no universal mechanistic convergence exists and syndrome-based enrollment does not map to a single causal pathway. In such settings, the risk of false RCT transport and population-level harm rises substantially because trial outputs are no longer anchored to a shared, transportable mechanism. These trials, including recent corticosteroid REMAP-CAP study [18], exemplify the classic Petty–Bone RCT discussed in detail elsewhere [1] and have, for decades, been near universally negative or reversed following harm associated with false RCT transport.

Importantly, a trial’s position along the cause agnostic RCT–causal integrity RCT spectrum is not a judgment of methodological rigor or analytic quality. Cause agnostic RCTs may be meticulously conducted, fully **C**onsolidated **S**tandards **o**f **R**eporting **T**rials -compliant [9], and statistically sound. The distinction concerns the causal question the trial can answer and whether its entry criteria and treatment results can be safely transported into guidelines and bedside protocols. Failure to recognize this distinction allows population triage to falsely substitute for causal unity, converting apparent trial success into a public-safety hazard.

Recognizing the cause agnostic RCT–causal integrity RCT spectrum at the design stage is therefore essential for a guideline committee while evaluating the level of the evidence for transportability, guiding protocol development, and preventing large-scale harm arising from design-level causal misalignment.

## Causal symbolic modeling

Causal symbolic modeling is a design-level causal framework that a clinician in cooperation with a statistician can use to interrogate whether the symbolic elements of a planned trial correspond to causally coherent entities capable of supporting safe transport to clinical protocols. Causal symbolic modeling was recently introduced as a simple means to interrogate cause agnostic RCT in a review of the cause of false RCT transport in critical care trials and the interested reader is directed to that review for examples of such interrogation [1]. Operationally, causal symbolic modeling simply formally applies the logic of structural causal modeling upstream of trial execution to explicate the causal integrity of design assumptions. Structural causal modeling is presented in “Causal Inference: What If” [30] and the “The Book of Why’ [31] but the clinician need only learn the most basic modeling to interrogate an RCT design as was accomplished using directed acyclic graphs in the referenced review [1].

Causal symbolic modeling is simply upstream structural causal modeling and it does not introduce new mathematics, replace randomization, or adjudicate causal truth from data. Rather, it formalizes the process of bringing clinicians and statisticians together before trial initiation to formalize causal explication and object validation. In other words it provides a simplified, missing formal bridge between RCT design theory and causal inference.

By interrogating disease labels, cohort gates, interventions, outcomes, and protocol rules, causal symbolic modeling determines whether downstream, randomization, and statistical estimation can meaningfully support safe transport of trial criteria. Table 3 shows the advantage of embedding this modeling into the **C**onsolidated **S**tandards **o**f **R**eporting **T**rials (CONSORT) [9] to increase the safety of RCT transport to clinical protocols. The appendix below provides a transport safety checklist for addition to this standard.

Because trial registration represents the first public declaration of causal intent, and the operationalization of causal intent is a patient safety concern, trialists should use causal symbolic modeling or another interrogation tool prior to registration. Explicit reporting of causal coherence and transportability assumptions would allow funders, reviewers, and clinicians to identify structurally unsafe designs early, reducing waste and harm.

**Table 3:** Proposed additions to CONSORT to increase causal integrity of the trial

CONSORT Item	CONSORT Requirement	What Structural Causal Modeling Adds
Item 12: Eligibility Criteria	Report inclusion/exclusion criteria	Validates causal coherence of participants
Item 13: Interventions	Describe interventions	Checks causal mechanism stability
Item 21: Statistical Methods	Describe analytics	Checks whether a meaningful, stable causal estimand exists
Item 30: Generalizability	Discuss external validity	Detects risk of false RCT transport

## Pragmatic trials

Pragmatic randomized trials are essential for evaluating how interventions perform under real-world clinical conditions. When used appropriately, they test the robustness, scalability, and safety of treatments whose causal targets are already established. Problems arise not from pragmatism itself, but from deploying pragmatic designs in the absence of causal integrity. Without prior confirmation that population membership reliably selects a shared causal treatment target, pragmatic trials risk amplifying false RCT transport by accelerating dissemination of population-based treatment protocols that lack causal alignment. Pragmatic trials are therefore most effective when positioned downstream of causal-integrity evidence, not as substitutes for it.

## Mapping the Continuum: Pure CIR → Pragmatic CIR → Unsafe Pure CAR

### • Pure Causal-Integrity RCT (CIR)

Treatment targets a specific causal disease mechanism. Patient selection uses a diagnostic test that reliably selects that mechanism. Entry criteria and treatment logic are transportable to bedside.

### • Pragmatic CIR

Causal target remains defined and coherent, but selection criteria are broadened to test effectiveness across real-world settings. Pragmatism evaluates durability and implementation without abandoning causal alignment.

### • Unsafe Pure CAR

No reliably transportable selection defining a shared causal treatment target. Broad disease and cause agonistic population triage alone defines eligibility. Randomization substitutes for causal coherence, and trial entry criteria are highly vulnerable to false RCT transport when embedded into clinical protocols.

This progression illustrates that risk emerges not from pragmatism, but from loss of causal integrity.

## RCT failure mode “blindness”

The randomized controlled trial has been elevated to such a privileged epistemic status that structural causal failures of the trials themselves were not recognized as the source of false RCT transport during the COVID-19 pandemic. Instead, post-hoc explanations were largely confined to enumerations of “trial limitations,” a framing reinforced by **C**onsolidated **S**tandards **o**f **R**eporting **T**rials -style standards [9] that emphasize internal validity and transparency while remaining largely silent on causal transportability and downstream protocol safety. As a result, RCT-derived clinical protocols in critical care operate in a domain effectively insulated from deep failure-mode analysis.

This insulation is institutional rather than accidental. NIH trial governance and reporting norms provide no requirement for formal failure mode and effects analysis [32, 33] of RCT design, nor for real-time surveillance of critical care protocol performance once is RCT-derived guidance implemented at scale. In contrast to other high-risk systems governed by federal safety mandates, critical care medicine lacks structural safeguards to detect and halt flawed RCT structure and protocol malfunction, allowing flawed RCT and design-level causal errors to propagate into widespread public harm before correction occurs.

The COVID-19 episode exposes a false premise in RCT governance: that internal statistical validity is sufficient to guarantee clinical safety. In cause-agnostic RCTs, enrollment thresholds and intervention rules can be internally valid yet causally misaligned with the treated population, rendering protocol failure structurally predictable once transported into practice. Despite early signals of implausible mortality, physiologic discordance, and clinician abandonment, cause agnostic RCT-derived ventilator protocols persisted during COVID-19 because no requirement existed for pre-deployment failure mode and effects analysis or real-time protocol surveillance. These protocols were ultimately abandoned not through formal institutional review or new randomized evidence, but through clinician recognition of physiologic incompatibility demonstrating that the failure lay not with individual physicians, but with the absence of system-level safeguards against false RCT transport.

## Conclusion

We conclude that the “evidence deployment paradox,” in which apparently strong evidence of benefit reverses to harm when trial entry criteria are transported into clinical treatment protocols, arises from a four-decade deviation in RCT design within critical care. This deviation replaced disease diagnosis and causal specificity with consensus-based, broad disease- and cause-agnostic triage thresholds as trial inclusion criteria. While this approach streamlined trial implementation, it compromised the trial’s causal integrity and its safe transportability to clinical guidelines.

Preventing future RCT based, guideline associated public harm will require reform of the potentially unsafe cause-agnostic randomized controlled trial. The guideline committee should identify the state of causal integrity of an RCT and adjust the level of the evidence, as well as the safety and scope of transportability accordingly. Achieving this will require investment in an infrastructure that educates clinicians, trialists, grant reviewers, and members of consensus and guideline panels in the fundamentals of structural causal modeling. To enhance public safety, basic structural causal modeling should become a design requirement for RCT grant applications. Such training need not be extensive, as the foundational principles of causal modeling relevant to RCT design are conceptually straightforward and can be readily incorporated into existing reporting standards and research training programs.

## Supplementary Information

Below is the link to the electronic supplementary material.

Supplementary Material 1

## Data Availability

No datasets were generated or analysed during the current study.

## Glossary of Terms

Causal-integrity Randomized Controlled Trial (CIR): A randomized controlled trial designed to answer a causal question by aligning enrollment, intervention, and outcome with a disease mechanism or etiologic target. Causal Integrity RCT preserve causal coherence and support principled safe transport of results to patients who share the same causal structure.
Cause-Agnostic Randomized Controlled Trial (CAR): A randomized controlled trial in which enrollment is defined by syndromic or threshold-based criteria rather than by a causally diagnostic disease mechanism. CARs may achieve internal validity within the enrolled population but may lack the causal specificity required for safe transport into generalized clinical protocols.
Evidence Deployment Paradox: Strong evidence of benefit from robust RCTs or meta-analyses followed by unexpected harm during clinical application despite the use of the same timing and setting of treatment as well as the same treatment criteria used in the trial.
False RCT Transport: The application of trial-derived protocols or conclusions to patients who do not share the causal structure represented in the source trial population. False RCT transport arises from structural design features rather than analytic error or misuse of data.
Institutionalized Causal Agnosticity: A institutionalized trial design stance in which the causal structures linking intervention, mechanism, and outcome are not specified or encoded (e.g., no explicit causal model or directed acyclic graph), and population-level outcome differences are treated as sufficient evidence of efficacy. Although such designs may preserve internal validity, the absence of causal structure substantially increases the risk of false RCT transport when trial protocols are transported to a clinical population.
Institutionalized Cause Agnostic Trial Lineage: The historical embedding of standardized cause-agnostic trial design within NIH-supported research networks, consensus panels, guideline development, and quality frameworks.
Symbol–mechanism substitution fallacy: The false assumption in synthetic-syndrome reasoning, that fulfillment of syndrome consensus criteria alone is sufficient to justify “strong” evidence for a treatment protocol transport to a patient regardless of whether that patient’s disease which is targeted by the treatment protocol was sufficiently represented in the source trial or meta-analysis or even existed at the time of the source trial.
Syndromic Enrollment Gate: A consensus-derived set of physiologic thresholds or composite criteria used as triage for trial eligibility in the absence of causal diagnosis. Such gates function as selection devices within trials but can cause the “evidence to deployment paradox” and harm when transported to clinical protocols
Trial Production Centered Governance: A research governance orientation in which success is defined by enrollment efficiency, statistical output, protocol adherence, and trial production rather than by causal discovery, transportability, reproducibility, and buildable knowledge construction.
RCT Failure-Mode Blindness: The absence of formal mechanisms to detect or respond to RCT generated protocol-level failure or harm arising from design-induced false RCT transport, under emergency conditions or later in formal retrospective review.
