# Supplementary material for: Inherent safety risks of guidelines derived from “cause-agnostic” randomized controlled trials: a review
Source: Patient Saf Surg. 2026 Apr 16;20:22. doi: 10.1186/s13037-026-00486-y (PMC13244930; doi:10.1186/s13037-026-00486-y)
Supplement: Supplementary file 1 — Supplementary Material 1 [file 13037_2026_486_MOESM1_ESM.docx]

**Appendix: RCT Transport Safety Checklist**

**A. Disease/Cause Definition**
• Is the disease/cause label causally coherent or syndromic?
• Are multiple different diseases aggregated symbolically?

**B. Trail Entry Gate (enrollment criteria)**
• Is the entry gate a disease and cause agnostic triage?

**C. Intervention–Disease Alignment**
• Does the intervention target a shared mechanism?

**D. Transportability**
• Would effects persist if disease mix changed, despite still meeting the entry criteria?
